# Effect of sugar beet variety resistance on the disease epidemiology of *Cercospora beticola*

**DOI:** 10.1002/ps.8666

**Published:** 2025-01-23

**Authors:** Yixuan Yang, Mark Varrelmann, Sebastian Liebe

**Affiliations:** ^1^ Department of Phytopathology or Coordination Institute of Sugar Beet Research Göttingen Germany

**Keywords:** *Cercospora beticola*, disease epidemiology, variety resistance, sugar beet, spore flight

## Abstract

**BACKGROUND:**

*Cercospora* leaf spot (CLS), caused by *Cercospora beticola*, is the most destructive foliar disease in sugar beet. CLS is conventionally controlled with fungicide, but the emergence of fungicide‐resistant populations reinforces the importance of developing and cultivating resistant varieties. Understanding the dynamics of CLS in different varieties is hence essential for sustainable CLS management.

**RESULTS:**

Field experiments (2022 and 2023) with four sugar beet varieties possessing different resistant properties were conducted to describe the relationship between the variety resistance and the disease epidemiology of *C. beticola*. For this purpose, spore flight and disease progression were assessed on a weekly basis. Disease severity (DS) and disease incidence (DI) were delayed in resistant varieties compared to the susceptible and moderately susceptible ones. This finding was further confirmed by a model‐based analysis of DS and DI for all varieties. Weekly spore flight monitoring during the vegetation period showed a similar tendency of reduced spore quantity by the resistant varieties. This was probably due to the lower DS, as no differences were found when the amount of fungal DNA was determined in individual lesions from the different varieties. Analysis of relative yield loss further confirmed the advantage of growing resistant varieties.

**CONCLUSION:**

Our results highlight that resistant varieties delay disease onset resulting in less severe symptoms and reduced spore flight. We also proved that aerial spore flight intensity could reflect the resistant property of each variety. These results provide a deeper insight into the interaction between variety resistance and CLS epidemiology, emphasizing variety‐specific CLS management. © 2025 The Author(s). *Pest Management Science* published by John Wiley & Sons Ltd on behalf of Society of Chemical Industry.

## Graphical abstract

*Cercospora* leaf spot (CLS), caused by *Cercospora beticola*, is the most destructive foliar disease in sugar beet. In our two‐year field trial, we monitored the spore flight of *C*. *beticola* in field trials comprising varieties with different resistant properties with a quantitative polymerase chain reaction (qPCR)‐based method.

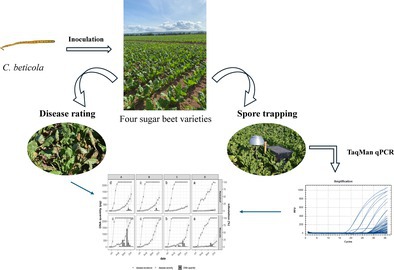

## 1 INTRODUCTION

*Cercospora* leaf spot (CLS) is the most destructive foliar disease in sugar beet,^1^ which was first described by Saccardo.^2^ CLS infected sugar beets exhibit gray‐centered circular necrotic lesions on leaves and petioles. As infestation progresses, a growing number of lesions leads to destruction of the leaves and petioles, and later to a defoliation of the whole plant.^3^ The economic loss caused by CLS in sugar beet production affects the root yield, and more importantly the recoverable sucrose.^4^, ^5^ Additionally, extra economic input is required in the further processing of sucrose recovery and extraction from CLS‐infested sugar beet.^6^

The causal agent of CLS is the fungal pathogen *Cercospora beticola*, which belongs to the phylum of Ascomycota. Its polycyclic lifestyle allows multiple cycles of infection during a growing season.^4^, ^7^ In natural environments, the infection is initiated by the pseudostromata present on plant debris during the winter. The pseudostromata subsequently produce conidia‐type spores that can be dispersed by wind, water splash and human activities.^8^ Once the spores successfully attach on the surface of leaves, they penetrate through stomata and develop hypha intercellularly. The formation of the lesions is a sign of switching to its necrotrophic phase.^9^ As infestation progresses, more pseudostromata are established from the center of the lesions, contributing to the later infection cycles. Therefore, the devastating epidemics of CLS are the consequence of the repetitive sequence of infections.^7^

Similar to many other plant pathogens, *C. beticola* favors a high relative humidity (RH) and temperature for conidia production.^5^, ^10^ ^,^ ^11^ Food and Agriculture Organization of the United Nations (FAO) has addressed the potential risk of pathogen outbreak coupled with climate change.^12^ Therefore, a higher risk of *C. beticola* epidemics can be expected. In Germany, the CLS epidemic is no longer restricted to southern and western regions. A shift of CLS epidemic towards northern and eastern region was observed by Vogel *et al*.^13^ A CLS risk simulation study conducted by Kremer *et al*. predicted an increased risk and an earlier occurrence of CLS due to climate change in Germany.^12^, ^14^

Various approaches have been applied to control CLS in agriculture, including agronomic practices, fungicide applications, and utilization of host resistance, in which fungicide application is still the key manner in CLS management.^15^ In practice, fungicide groups such as demethylation inhibitors (DMIs), quinone outside inhibitors (Qols), methyl benzimidazole carbamates (MBCs) and succinate‐dehydrogenase inhibitors (SDHIs) are the most commonly applied in fungal pathogen management.^16^ Nevertheless, reported fungicide resistance in some *C. beticola* populations in Germany^16^ highlights the potential risk of reduced efficacy of fungicides. Hence, research in other disease management approaches in necessary for integrated pest management (IPM) of *C. beticola*.

Selective breeding has been applied to enhance host resistance to plant pathogens with different natures of resistant mechanisms.^17^ The first monogenic resistance to *C. beticola* in sugar beet was identified by Lewellen and Whitney.^18^ The corresponding variety was soon abandoned due to the instability of the resistance.^19^ By reason of the high allelic diversity in wild relatives in sugar beet, they are frequently used as genetic resources for *C. beticola* resistance in cultivated species.^17^, ^19^ Some wild relatives of sugar beet exhibiting significant resistance against *C. beticola* attract substantial scientific attention. Sea beet (*Beta vulgaris* spp. *maritima*) contributes to the first success in introducing resistance to sugar beet.^19^, ^20^ Quantitative trait locus (QTL) analysis performed by different researchers suggested that four or five QTLs are responsible for CLS resistance.^21^, ^22^ Subsequently, a more comprehensive investigation on CLS resistant QTLs was carried out by Taguchi *et al*.,^19^ revealing two particular QTLs strongly responsible for CLS resistance. However, up to date, no resistant variety of sugar beet was able to completely avoid being infected by *C. beticola*. Instead, a delayed disease onset and a reduced disease progression were observed on some resistant varieties.^15^, ^23^

Previous studies have been focused independently on exploring host resistance, spore flight intensity or CLS epidemiology. However, scientific support for the interaction between these aspects remains limited, although it is important as it has an impact on disease management with fungicides. Hence, the objective of our study was to describe the impact of the sugar beet variety resistance on *C. beticola* spore flight and epidemiology. For this aim, we evaluated aerial spore production and disease development of four sugar beet varieties with distinct resistant properties in 2022 and 2023.

## 2 MATERIALS AND METHODS

### 2.1 Field trial design

To demonstrate the impact of variety resistance on spore flight, field trials were carried out in two consecutive years from 2022 to 2023 in four geographical locations near Göttingen, Germany. In 2022, trials were performed in Weende (51°34′04.2″ N 9°54′45.1″ E) and in Reinshof (51°29′57.8″ N 9°54′58.9″ E), while in 2023 Weende Große Lage (51°33′53.6″ N 9°54′58.3″ E) and Marienstein (51°36′26.1″ N 9°55′06.2″ E) were selected for the trials. In both years, four sugar beet varieties, namely variety A (susceptible), variety B (susceptible), variety C (resistant), and variety D (resistant), were cultivated in triplicates as a completely randomized block design in each location. The genetic background and the nature of resistance of each variety were not known. Inoculation was done manually with 4 g/m^2^ of CLS infected leaf material as described previously.^4^ The inoculum was prepared by blending the air‐dried, CLS‐infected sugar beet leaf material with semolina in a 1:5 ratio.^4^

Varieties of interest were cultivated as six‐row plots and inoculated artificially after canopy closure. In both years, inoculated plots were adjacent to non‐inoculated border rows of resistant variety C to avoid cross contamination. In addition, non‐inoculated control plots for all four varieties were incorporated to determine the relative yield loss in 2023. The control plots were cultivated in duplicates and treated with fungicide to prevent CLS. Three application sessions were conducted with different schemes (Supporting Information Table S1).

### 2.2 Disease assessment

To evaluate the disease development on each variety throughout the growing season, disease assessment was performed at weekly intervals in both locations for all plots. Disease severity (DS) and disease incidence (DI) are the two parameters used for disease assessment in this study. For this, 36 sugar beet plants were randomly selected from each plot for assessment. DS was recorded as the symptomatic leaf area (in %) of the middle leaf of a chosen plant, while DI was defined as the total number of the sugar beet individuals that showed symptoms in an examined population at the time of monitoring. Disease assessment began 1 week post‐inoculation (wpi) and continued until harvest.

### 2.3 Modeling of the disease development

To gain a deeper insight into epidemics of each variety in different locations from both trial years, we modeled the DI and DS separately for further comparison among varieties. We implemented a total number of 32 models for both DI and DS. Each model was fitted to describe the specific epidemic of CLS of each variety from each location in each year. By visualizing the disease development curves, we safely assumed that all curves follow a sigmoid curve which can be described by either a logistic model^24^ or a Gompertz model.^25^ Due to the rapid increase of DI at the beginning of the season, the epidemic curve tended to be asymmetric. Therefore, we used Gompertz model to describe the DI development, while the DS was modeled based on a logistic curve.

The equations for both models are described as:

(1) $$\mathrm{DS}=\alpha /\left(\left(1+\exp\left(\beta -\gamma \times \mathrm{wpi}\right)\right)\right)$$

(2) $$\mathrm{DI}=\alpha \times \exp\left(-b2\times b3^{\mathrm{wpi}}\right)$$

Equation (1) describes a logistical development of DS over the monitoring time (wpi). The *α* is the upper asymptote (the maximum possible value of DS which maximizes the model fitting). The *β* represents the parameter that shifts the logistic curve along the wpi. The *γ* is the growth rate parameter. Equation (2) represents how the DI developed following the Gompertz curve along the wpi. The *α* is the upper asymptote (the maximum possible value of DS which maximizes the model fitting); *b*2 is the parameter that determines the rate at which DI declines; *b*3 is the parameter that determines the growth rate of DI along the wpi. Considering that fungicide application in Germany is based on the summary threshold system,^26^ we further selected these thresholds of DI (5%, 15%, and 45%) to calculate the corresponding wpi for each model.

All steps for model implementation and model fitting were conducted in R (version 4.4.0, R Core Team 2021). We extracted data of each variety from each location in each year, resulting in 32 datasets. We first calculated the initials of each model by applying function *getInitial*() to a *selfStart*() logistic model or Gompertz model in package stats with corresponding dataset. Models were fitted with function *nls*() and the coefficient of determination (*R* ^2^) was later calculated to examine how well the models fit the corresponding data. In the case when *getInitial*() function was not able to calculate the initials due to the unique feature of certain datasets, initials were assigned manually and then fitted the models using *nlsLM*() function in package *minpack.lm* (version 1.2‐4).^27^ All final initials were determined after the model fitting.

### 2.4 Spore flight detection and quantification

A method that is able to determine the spore flight intensity in field plots was established at the Institute of Sugar Beet Research, Göttingen, Germany by Imbusch *et al*.^4^ In our study, we used the same approach with minor modifications to confine and quantify the spore flight intensity in our field trials. In summary, Roto Spore Samplers (Agri Sampler Ltd, High Wycombe, UK) (Supporting Information Fig. S1) were situated 50 cm above the ground on supportive poles at the middle of the inoculated plots. More specifically, spore samplers were installed at the center between the third and fourth row of the plots. All replicates of three varieties were selected for spore flight monitoring in each location each year. The motor of the sampler was attached with two replaceable rods (approximately 5 cm in length), covered with Vaseline spray. The rotor spins at 3500 rpm to capture airborne *C. beticola* spores. The sampler was programmed to spin every day from 00:00 to 23:58 with 2 min run/stop intervals. Spores‐embodied rods were sampled at weekly intervals on the same day as disease assessment. Sampled rods were immediately stored in DNeasy PowerLyzer PowerSoil bead tubes supplied along with the kit (Qiagen, Hilden, Germany) for further DNA extraction. DNA extraction was done with the aforementioned kit, following the supplied protocol with an additional heating step (10 min at 70 °C) after homogenizing the samples. DNA samples were verified for integrity through gel electrophoresis. DNA quantity was examined with a spectrophotometer (DS‐11 Series; DeNovix Inc., Wilmington, DE, USA).

Extracted DNA samples were further quantified through TaqMan Real‐time polymerase chain reaction (qPCR) assay; the same primers and probe were used as described by Imbusch *et al*.,^4^ targeting the *calmodulin* gene in *C. beticola*. The qPCR was executed with the CFX Real‐Time PCR Detection System (Bio‐Rad Laboratories, Hercules, CA, USA) in 96‐well plates. The details of the reaction mix and program was described by Imbusch *et al*.^4^ Samples from each plot collected from the same time point were examined in triplicates on one plate. The qPCR assay was repeated if all three technical replicates of one sample exhibited significantly variable results. Prior to the season start, a pure culture of *C. beticola* was prepared to obtain the qPCR standards. For this, a 2‐week old mycelium plug from the pure culture was cut and subsequently cultivated in potato dextrose broth (PDB) for approximately 10 days. Fungal cell material was later harvested and lyophilized for DNA extraction. DNA extraction for fungal cell material was done with a DNeasy Plant Mini Kit (Qiagen). The extracted DNA sample served as the initial stock solution for the qPCR standards. The standards were generated through a serial dilution of the DNA stock described earlier with a dilution factor of ten. A total of five dilutions of standard DNA were generated for each qPCR reaction. The DNA quantity of each reaction was automatically calculated by the Bio‐Rad CFX Manager software (Bio‐Rad Laboratories) and further converted to picogram per sample manually for data analysis.

### 2.5 Yield and relative yield loss

To gain a better insight into the performance of different varieties after the CLS infestation, relative loss of white sugar yield (WSY) was evaluated in 2023. Beet brei was prepared and analyzed according to routine methods of the sugar industry [ICUMSA (International Commission for Uniform Methods of Sugar Analysis), 2007]. For the data from 2023, relative loss (%) of WSY of each variety in both trial locations were calculated with Eqn (3).

(3) $$\text{Relative loss}=\left(1-\frac{{\text{Yield}}_{\text{inoculated}}}{{\text{Yield}}_{\text{control}}}\right)\times 100\%$$

### 2.6 Glasshouse inoculation assay

To quantify *C. beticola* DNA of individual lesions from different varieties, a glasshouse inoculation assay was performed on all our four varieties of interest. Eight plants from each variety were transplanted to single pots a week after germination. Inoculation was performed 6 weeks after transplanting. Six of eight plants from each variety were artificially inoculated with *C. beticola* conidial suspension, while the other two plants were kept healthy as control. For inoculation, spore suspension was acquired from single‐isolate‐infected leaf materials which was preserved at −20 °C as stock inoculum. Leaf materials were incubated in Petri dishes (∅ 90 mm) with wet filter papers covered on the bottom of the Petri dishes, sealed, and kept in darkness 4 days prior to inoculation. After incubation, sporulation of *C. beticola* on the leaf material was confirmed under a stereomicroscope. Leaf material from each Petri dish was further rinsed with water with added glass beads in a 50 mL centrifuge tube. The suspension was filtered with a sieve to remove the excessive leaf material and glass beads. The quantity of the infectious units (IUs) was defined by counting under the microscope with a Neubauer hemocytometer, and a final concentration of 30 000 IU/mL was determined. An amount of 7 mL of the spore suspension was misted with a glass sprayer on each plant. Inoculated plants were covered with a plastic foil for 6 days under a consistent temperature of 30 °C with a 12‐h photoperiod. Three‐day‐old lesions were sampled for fungal DNA quantification. Specifically, newly emerged lesions were marked on a daily base. Three days after emergence, lesions with similar size were sampled with a cork borer (hole size ∅ 7.5 mm). Harvested lesions were immediately stored in liquid nitrogen and further preserved at −20 °C until DNA extraction. Thirty lesions from each variety were used for DNA extraction and biomass quantification. DNA was extracted by DNeasy Plant Mini Kit (Qiagen) with an elution volume of 60 μL. The integrity of the DNA was examined by gel electrophoresis to ensure no degradation occurred during handling. The fungal DNA of *C. beticola* on each single lesion was quantified by the TaqMan® qPCR assay described earlier.

### 2.7 Statistical analysis

Regarding the disease development data, the mean DS from a population of 36 sugar beet plants from each plot at each monitoring time point was initially calculated prior to the statistical analysis. DI was calculated from the number of infested individuals divided by the total number of examined plants of all three replicates. Data processing and statistical analysis were performed using the program R. A calculation of the area under the disease progress curve (AUDPC) was performed by *audpc*() function in *agricolae* ^28^ and *dplyr* package.^29^ DS data were used for statistical analysis of disease development on each variety. A generalized least square model with autocorrelated errors was used for analysis of variance (ANOVA).^30^ *Post hoc* analysis by a multiple mean comparison with the function *emmeans*() in the *emmeans* package^31^ was performed. Pairwise comparisons were adjusted using the Tukey method. The comparisons were further classified into homogeneous groups using *multicomp* package.^32^

To compare if different varieties showed differences in time to reach to certain DI and DS threshold values, we compared wpi values when DI reached to a threshold value 5%, 15%, and 45%. Similarly, we also analyzed the differences of wpi when DS reached 10%, 20%, and 30%. Calculated wpi were analyzed separately for DS and DI. Varieties from the same year and location were grouped for statistical analysis. For this, we performed model‐specific bootstrapping for confidence intervals assisted by *nlstools* package^33^ for the calculated wpi values at the corresponding DI or DS threshold values. Resampling was done 1000 times and the corresponding wpi values were calculated. After assessing the normality of bootstrapped data, a Kruskal–Wallis test was performed followed by a Dunn's test for pairwise comparison.

In terms of spore flight quantification, the cumulative quantity of spores captured throughout the whole season in each plot was calculated for statistical analysis. The function *lm*() was used to apply a linear model. Residual normalization was done by log transformation. ANOVA and *post hoc* analyses were conducted as described earlier. Pairwise comparisons were adjusted using the Sidak method. The comparisons were further classified as described earlier. Statistical analysis of the yield data was performed with the aforementioned methods.

For the quantification of *C. beticola* fungal DNA in a single lesion, the mean values of the three technical repetitions of each sample in the qPCR assay was calculated for further statistical analysis. The function *lm*() was used to apply a linear model. ANOVA and *post hoc* analyses were performed as described. Pairwise comparison was adjusted using the Tukey method.

Levene's test and Shapiro–Wilk test was conducted before ANOVA in all cases for checking the variance homogeneity and normality. Data visualization in this study was done with the R package *ggplot2*.^34^

## 3 RESULTS

### 3.1 CLS development and spore flight intensity in 2022

**FIGURE 1:**
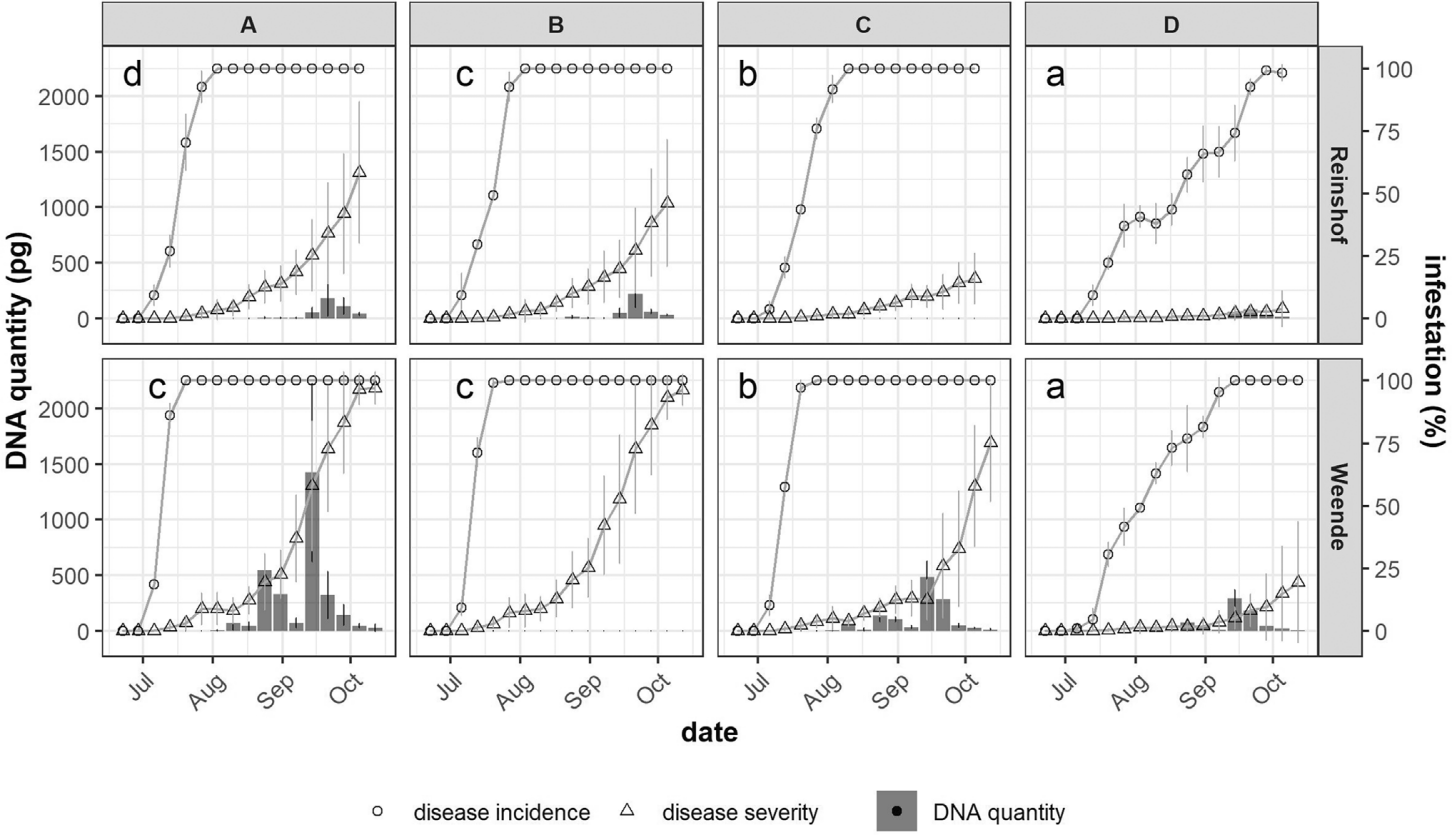
Mean value of disease incidence (DI) and disease severity (DS) (*n* = 108) and mean value of detected *Cercospora beticola* DNA (pg) (*n* = 3 plots) in 2022. For spore flight monitoring, varieties A, B, and D were selected for location Reinshof, while varieties A, C, and D were selected for location Weende. Error bars of DS data represent the standard deviation of three replicated plots of each variety. Error bars of *C. beticola* DNA represent the standard deviation of three field plots of qPCR. Small letters indicate the significant differences in DS among varieties (*P* < 0.05).

In 2022, weekly CLS development and spore flight intensity was monitored in trial locations Reinshof and Weende. Although our monitoring started immediately 1 wpi, symptoms were not observed until the third week after the inoculation in both locations (3 wpi). Symptoms that were at early stage displayed small *C. beticola* lesions on limited number of plants from all varieties in both trial locations. In both locations, varieties A and B reached DI of 100% the earliest whereas variety C showed a 1‐week delay (Fig. 1). CLS was not able to infect all sugar beet plants in all three plots of variety D until the late season reflected by the drastic delay to reach 100% DI. From the aspect of DS (Fig. 1), CLS progressed slowly in the early season from July to mid‐August for all varieties in both locations. However, starting from mid‐August, a rapid disease progress was observed in varieties A and B, while this sharp increase of DS was not observed until mid‐September in variety C in both trial locations.

In Reinshof, we found that all four varieties showed significant differences in DS. At the end of the season, susceptible varieties (A and B) were able to reach an average DS more than 45%, however, DS on resistant varieties was below 20%. Variety D particularly remained at an average DS even less than 5%. In Weende, with a higher infestation pressure, we observed no significant difference between varieties A and B, but in varieties C and D. A sharp increase in DS was observed earlier in susceptible varieties starting from the end of August. However, this sharp growth of DS started approximately 1 month later in resistant varieties. At the end of the season, despite the late increase, DS in variety C still reached 75%. However, variety D only showed an average DS less than 20% at the end of the season.

*Cercospora beticola* spores were captured and quantified during the season at the same date of disease assessment (Fig. 1). The first detection of aerial spores occurred at 7 wpi from the susceptible variety A in Weende (Fig. 1). In this location, we observed a significantly more intensive spore flight in variety A, followed by varieties C and D. Two spore flight peaks were observed in late August and mid‐September from varieties A and C. One peak of spore flight from variety D was observed in mid‐September, occurred together with the second peak from varieties A and C (Fig. 1). In Reinshof, a comparable amount of *C. beticola* DNA was perceived from varieties A and B. Spore flight intensity peaked at the end of September for both varieties, which aligned to the time when a sharp increase in DS was observed. Nevertheless, a small amount of *C. beticola* DNA was detected from variety D (Fig. 1).

**FIGURE 2:**
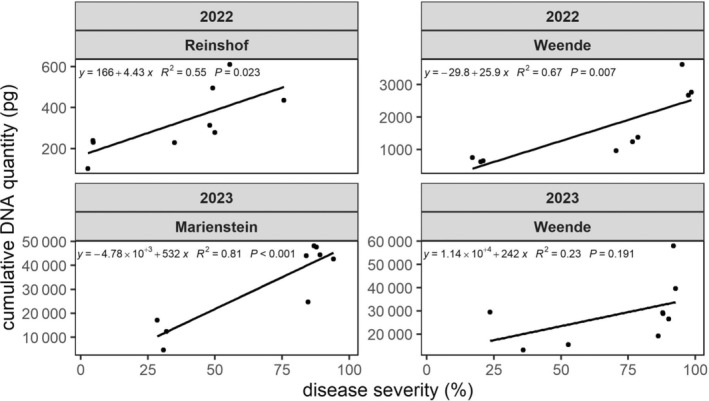
Correlation between cumulative DNA quantities in picograms detected from *Cercospora beticola* aerial spores and the disease severity (DS) at the corresponding time point. Data used for the correlation analysis in 2022 originated from the last sampling and rating time point. Data used for the correlation analysis in 2023 originated from the sampling and rating time point preceding the observation of severe regrowth [weeks post‐inoculation (wpi) = 13, corresponding to 9 June 2023].

We further calculated the mean value of cumulative *C. beticola* DNA quantity of each variety in each location (Table 1). The overall detected *C. beticola* DNA in Weende was substantially higher than in Reinshof (Table 1) following the same pattern as DS curves (Fig. 1). In Weende, we detected significantly less *C. beticola* DNA in variety C (1190.0 pg) and variety D (671.4 pg) compared with variety A (3018.1 pg). However, no significant differences in spore detection could be observed in Reinshof among different varieties (Table 1). In addition, a significantly positive correlation between DS and cumulative *C. beticola* DNA quantity was detected at both locations (Fig. 2).

**TABLE 1:** Comparison of area under the disease progress curve (AUDPC) and spore quantity in 2022 and 2023^†^, ^‡^

Year	Location	Variety	DS^§^ (%)	AUDPC	Spore quantity^¶^ (pg)
2022	Reinshof	A	58.28^d^	194.8^a^	402.7^a^
		B	46.19^c^	162.9^a^	384.1^a^
		C	15.85^b^	70.3^b^	—
		D	3.94^a^	15.1^b^	190.3^a^
	Weende	A	97.08^c^	480.8^a^	3018.1^b^
		B	96.39^c^	477.7^a^	—
		C	75.28^b^	223.8^b^	1190.0^a^
		D	19.39^a^	60.1^c^	671.4^a^
2023	Marienstein	A	87.64^c^	617.3^a^	45 570.3^a^
		B	87.96^c^	600.1^a^	56 002.9^a^
		C	72.5^b^	458.8^b^	—
		D	30.41^a^	191.7^c^	16 712.7^b^
	Weende	A	91.57^b^	698.6^a^	47 782.8^a^
		B	91.25^b^	658.0^a^	—
		C	87.41^b^	547.8^b^	31 331.9^a^
		D	37.45^a^	230.0^c^	25 996.7^a^

^†^ Lowercase superscript letters represent significant differences among varieties based on the adjusted Tukey's *post hoc* test (*P* < 0.05).

^‡^ Mean value (*n* = 3) of cumulative spore quantity.

^§^ In 2022, the disease severity (DS) of last rating time was shown. In 2023, DS before the regrowth [in weeks post‐inoculation (wpi)] was shown.

^¶^ —, Not selected for spore collection.

### 3.2 CLS development and spore flight intensity in 2023

**FIGURE 3:**
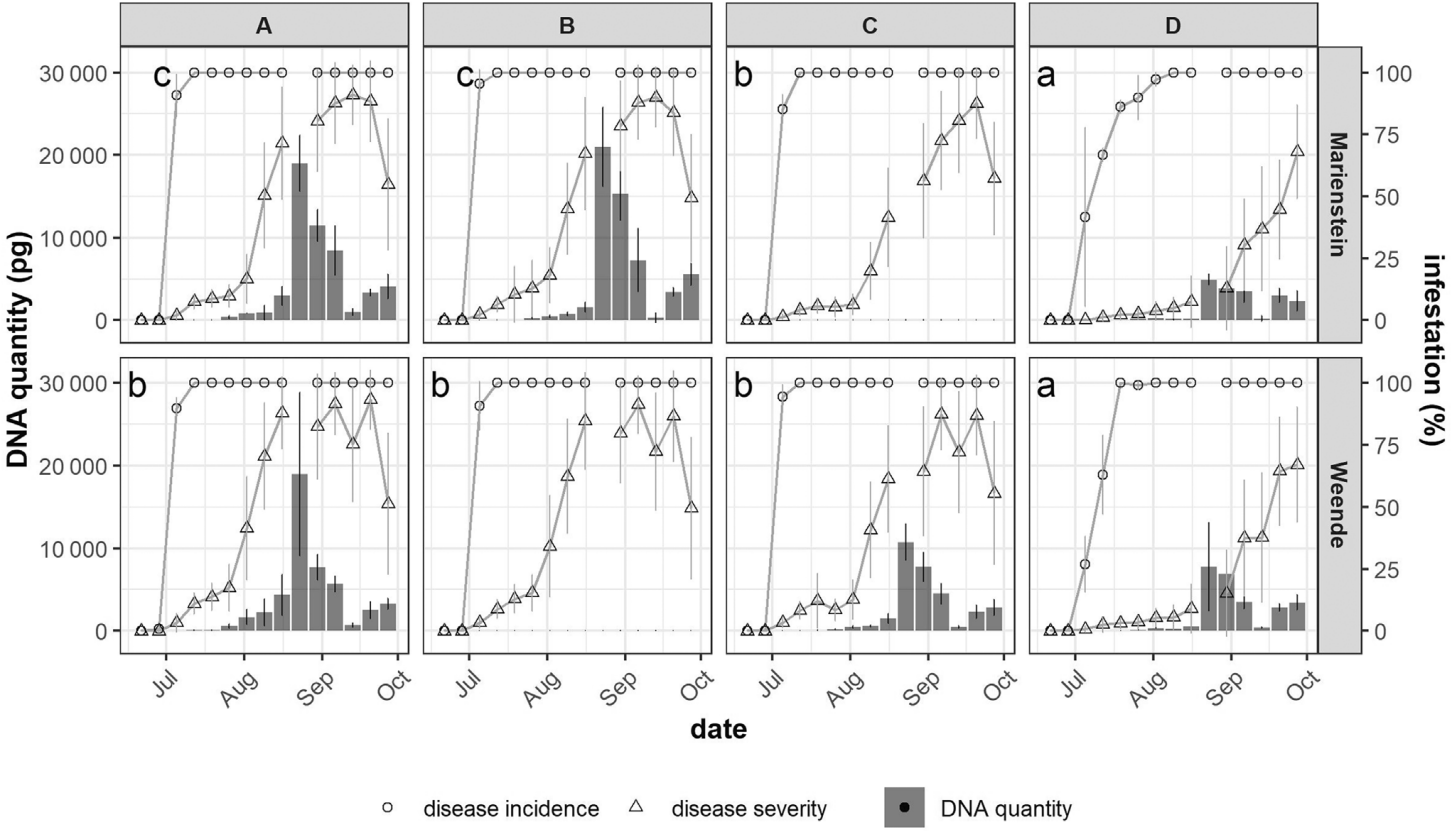
Mean value of disease incidence (DI) and disease severity (DS) (*n* = 108) and mean value of detected *Cercospora beticola* DNA (pg) (*n* = 3 plots) in 2023. DI and DS data at 11 weeks post‐inoculation (wpi) were not recorded. For spore flight monitoring, varieties A, B, and D were selected for location Marienstein, while varieties A, C, and D were selected for location Weende. Error bars of DS data represent the standard deviation of three replicated plots of each variety. Error bars of *C. beticola* DNA represent the standard deviation of three field plots of qPCR. Small letters indicate the significant differences in DS among varieties (*P* < 0.05).

The field trial was repeated in 2023 at locations Marienstein and Weende. Disease development and spore flight monitoring was conducted weekly starting from 2 wpi. At Marienstein, varieties A, B, and C reached 100% DI 5 wpi, while variety D approached to 100% DI with a 4‐week delay (Fig. 3). Significant difference in DS was observed among varieties bearing different resistance, while no difference was seen within the two susceptible varieties. DS started progressing quickly in varieties A and B from the end of July, while a rapid growth of DS in variety C started slightly later in this location. Variety D, however, did not present a notable increase in DS before the end of August. The reduction in DS observed in varieties A, B, and C was attributed to the regrowth of new leaves. Conversely, this phenomenon was not witnessed at all in variety D.

In Weende, varieties A, B, and C reached 100% DI at 5 wpi, followed by variety D with 1 week delay (Fig. 3). Furthermore, variety D showed a significantly lower DS compared with the other three varieties. A sharp increase of DS in varieties A, B, and C started from the end of July. However, this rapid growth was not observed until September for variety D. Moreover, the regrowth of new leaves was observed in varieties A and B at the end of August, which resulted in the reduced DS (Fig. 3). This occurrence was observed 2 weeks later in variety C, while no regrowth was observed in variety D.

Spore flight monitoring at both locations was performed in the same manner as in 2022. At both locations, we found that the spore flight intensified subsequently along with the elevated DS and peaked at 11 wpi (Fig. 3). In addition, spore flight could hardly be detected 3 weeks after the culmination in both locations from all varieties. In Marienstein, the initial detection of spore flight was recorded at 6 wpi in susceptible varieties along with the occurrence of the first increase of DS (Fig. 3). Even though spore flight peaked at the same time for all three monitored varieties, we detected nearly four times less *C. beticola* DNA in variety D than in susceptible varieties in this location. In Weende, the initial detection of spore flight occurred at 5 wpi in all varieties. At 11 wpi where the maximum of spore flight was reached, susceptible variety A showed approximately two times higher detected *C. beticola* DNA than resistant varieties. The cumulative amount of *C. beticola* DNA that was detected throughout the season was subsequently calculated. We noticed that the overall spore flight intensity in 2023 was markedly higher than in 2022 (Table 1). Significantly less spores were detected in variety D compared with susceptible varieties in Marienstein, while the varieties in Weende did not show significant differences compared with each other. Despite a positive correlation between DS and cumulative *C. beticola* DNA quantity can be pinpointed, this positive correlation was significant only in Marienstein (Fig. 2).

### 3.3 Model implementation for DI and DS

By fitting the DI and DS data of each variety in each location from each trial year, we implemented 16 Gompertz models and 16 logistic models to explain the development of DI and DS respectively (Table S2). High *R* ^2^ values of all models (≥ 95%) indicate an adequate goodness‐of‐fit of all models. We further grouped the models based on measures (DI or DS), years and locations to compare the differences among varieties in the same location and year.

In 2022 (Table 2), by comparing the time when DI reached certain threshold values (5%, 15%, and 45%), we observed significant delay of variety D reaching the threshold value of 15% and 45% in both locations. Variety C also exhibited significant delay in reaching 15% and 45% of DI but only in location Reinshof. In general, no significant difference between varieties A and B were observed in reaching these thresholds. Interestingly, variety D reached 5% DI the earliest in Reinshof based on the model estimation, however, this observation was not reproduced in location Weende. Meanwhile, the comparison when DS reached 10%, 20%, and 30% was only conducted in one location (Weende) when the DS reached 10% and 20%, in which both resistant varieties showed a significant delay to reach to the threshold values. As the infestation in variety D was never reached to 10% in Reinshof, the comparison among varieties could not be conducted. In the case of the other location Weende, the variety B was the earliest in reaching to all set thresholds, followed by varieties A, C, and D. Both resistant varieties displayed significant lag in reaching the 10% and 20% threshold of DS.

**TABLE 2:** Model‐based estimation of weeks post‐inoculation (wpi) in 2022 and 2023 when disease incidence (DI) or disease severity (DS) reached certain thresholds^†^

Year	Location	Variety	DI			DS^‡^		
			5%	15%	45%	10%	20%	30%
2022	Reinshof	A	3.27^a^	3.64^a^	4.33^a^	9.96	12.23	13.62
		B	3.10^b^	3.58^a^	4.47^a^	10.53	12.76	14.2
		C	3.37^a^	3.88^b^	4.85^b^	13.14	—	—
		D	1.69^c^	4.19^b^	8.41^c^	—	—	—
	Weende	A	2.75^a^	2.94^a^	3.29^a^	8.75^a^	10.22^a^	11.18
		B	2.88^ab^	3.10^a^	3.53^a^	8.47^b^	10.04^a^	11.06
		C	2.88^b^	3.16^a^	3.69^a^	11.27^c^	13.24^b^	14.38
		D	2.93^ab^	4.18^b^	6.50^b^	14.48^d^	16.99^c^	—
2023	Marienstein	A	3.11^a^	3.23^a^	3.45^ab^	7.24^a^	7.89^a^	8.33^a^	
		B	3.09^b^	3.19^b^	3.38^a^	6.82^b^	7.69^b^	8.29^a^	
		C	3.13^c^	3.26^c^	3.52^bc^	7.85^c^	8.88^c^	9.60^b^	
		D	2.86^d^	3.34^d^	4.21^c^	10.89^d^	12.30^d^	13.12^c^	
	Weende	A	3.08^a^	3.20^a^	3.44^ab^	6.24^a^	6.94^a^	7.42^a^	
		B	3.11^a^	3.23^a^	3.45^a^	6.44^b^	7.17^b^	7.66^b^	
		C	3.10^b^	3.20^b^	3.39^c^	6.97^c^	7.10^c^	8.55^c^	
		D	3.41^c^	3.76^c^	4.41^b^	10.51^d^	11.89^d^	12.70^d^	

^†^ Lowercase superscript letters represent significant differences among varieties based on the Dunn's test (*P* < 0.05).

^‡^ —, Unavailable data as DS did not reached to the thresholds.

In 2023, despite the significant differences among varieties in reaching 5% and 15% DI threshold, the differences were not pronounced until the threshold was set to be 45% (Table 2). Variety D lagged for approximately 1 week to reach to a DI of 45% compared with variety A in both locations. In the case of DS, unlike the scenario in 2022, all varieties were able to achieve the threshold values due to the higher infestation pressure. Variety B reached to all three threshold the earliest in Marienstein, while variety A was the earliest one reaching to all three thresholds in location Weende. Variety D showed significant delays in both locations to reach all three thresholds, highlighting the maximum of approximately 5 weeks delay reaching 30% DS.

### 3.4 Yield analysis

**FIGURE 4:**
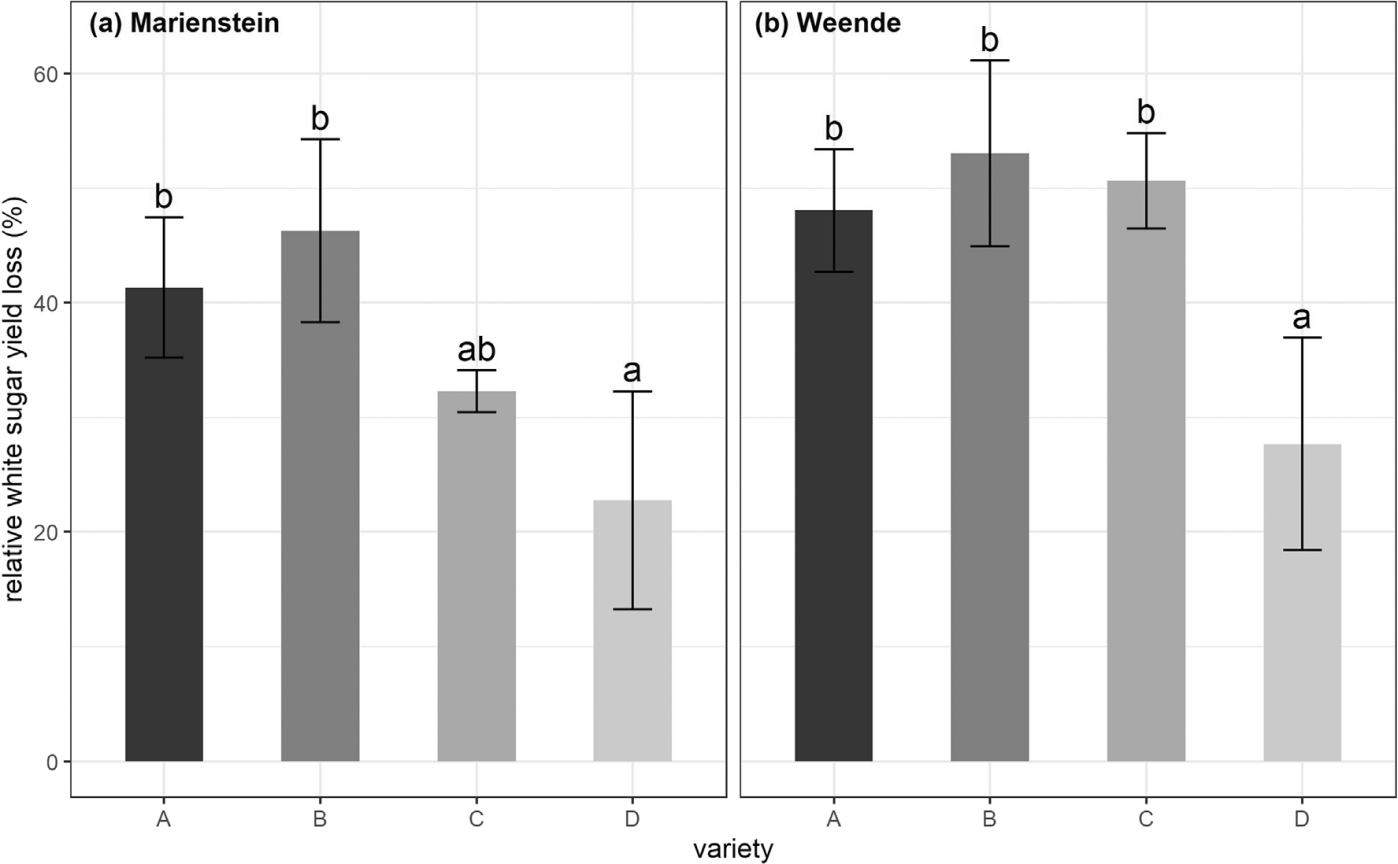
Mean value (*n* = 3) of relative loss in white sugar yield (%) in 2023 of four sugar beet varieties in (a) Marienstein and (b) Weende. Small letters denote the significant differences among varieties (*P* < 0.05). Error bars represent the standard deviation.

Relative yield loss of WSY of each variety were calculated in 2023. Significantly lower relative WSY loss in variety D was observed at both locations, while no significant differences were observed among varieties A, B, and C (Fig. 4).

### 3.5 *Cercospora beticola* DNA in single lesions on different varieties

**FIGURE 5:**
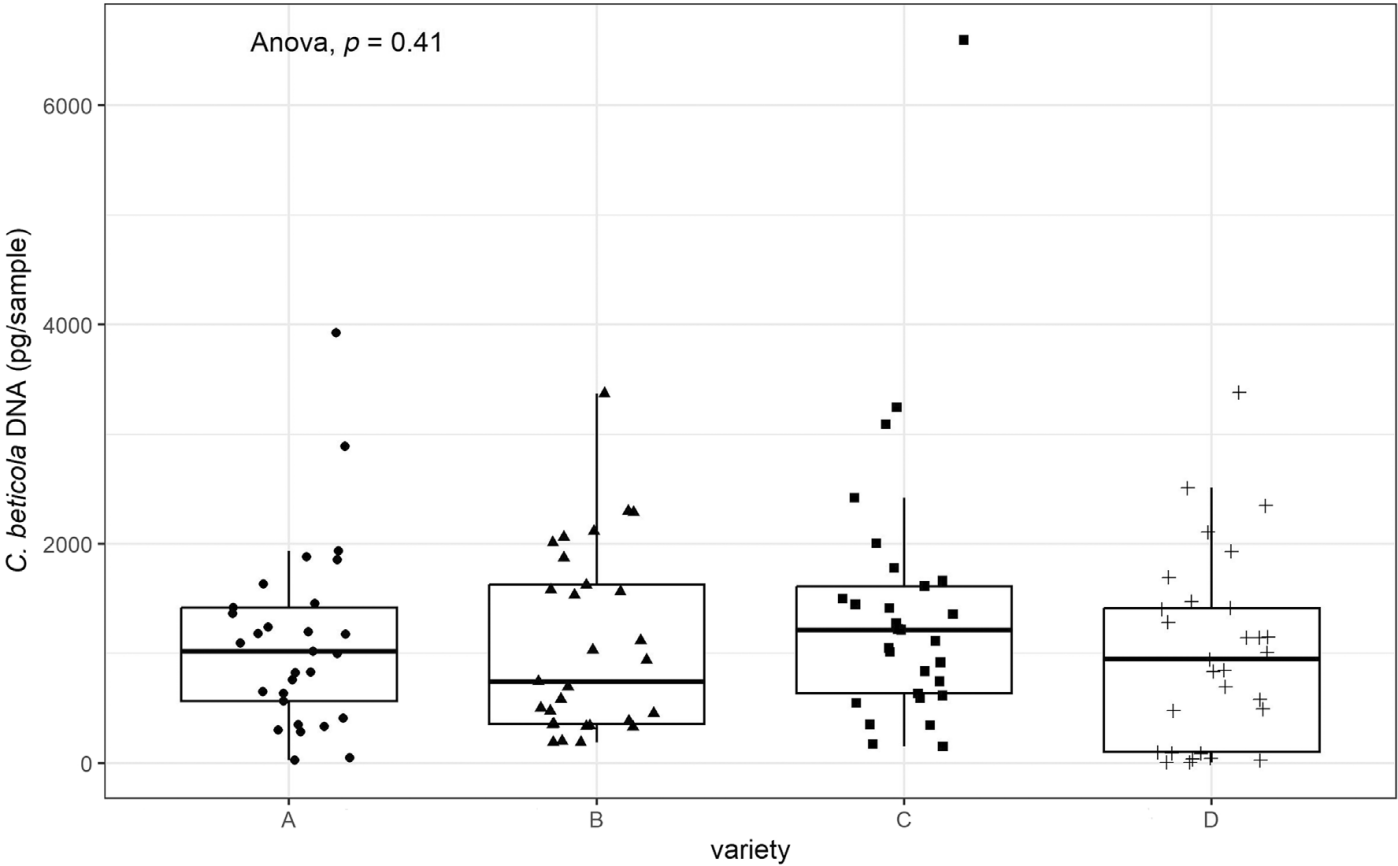
Boxplot of total DNA of *Cercospora beticola* on single lesion sampled from four different varieties (*n* = 30). Each jitter represents the mean value of three technical replicates of one sample.

The number of spores caught in plots from the resistant varieties was significantly lower in 2022 and 2023. The close correlation between DS and aerial spore production suggested that this effect was due to the lower DS of the resistant varieties rather than lower spore production per lesion. To provide further experimental evidence for this hypothesis, a glasshouse inoculation assay was conducted to quantify the fungal DNA of *C. beticola* in single lesions. For this purpose, 30 single lesions of the same age were collected from each variety and the fungal DNA was quantified by qPCR. The results (Fig. 5) revealed no significant differences in the detectable amount of *C. beticola* DNA in single lesions from the different varieties (*P* = 0.41).

## 4 DISCUSSION

Our results from disease rating over 2 years in different geographical locations confirmed that the four varieties of interest all convey different levels of susceptibility to *C. beticola*. Resistant varieties exhibited a delay in disease onset and a diminished severity compared to the susceptible ones. It must be pointed out that the artificial inoculation in our study provoked a high disease pressure which was necessary to guarantee sufficient DS for conidia production. However, this might have reduced the difference between varieties when environmental conditions were favorable for CLS. A markedly greater infection pressure of CLS was observed in 2023 considering the inoculation density remained the same as in 2022. In addition, a compromised resistance was observed in resistant varieties in 2023. This intensified infestation pressure can be the result of more favorable environmental conditions for CLS development. While high humidity can enhance the virulence of pathogens,^35^ certain environmental factors also affect the effectiveness of host resistance. Various defense mechanisms are deployed by plants to counter pathogen attacks; however, these mechanisms can be modified by environmental factors. For instance, the activation of PAMP‐triggered immunity (PTI) is claimed to trigger stomata closure which further blocks the entry of foliar pathogens.^36^ Nevertheless, it was shown that high humidity interrupts the stromata closure^37^ thereby allowing the penetration of pathogens.^38^ Compromised effector‐triggered immunity (ETI) associated with high relative humidity has been reported in the pathosystem of tomato (*Solanum lycopersicum*) and pathogen *Cladosporium fulvum*.^39^

Our findings in evaluating disease development were further confirmed by implementing variety specific models and compare the differences of early‐stage DI and DS development among varieties. By raising the thresholds, pronounced differences among varieties conveyed distinct resistant properties of varieties. Particularly when the threshold was set to be 45%, the delay in highly resistant variety D was up to 4 weeks. This finding is aligned with the study by Kaiser *et al*.,^23^ in which the difference among varieties was increased trending with the DI threshold. These results suggested that at least one of the resistant varieties was able to significantly delay the onset of CLS at the early stage of disease development. Yet, smaller values in variety D at 5% DI were occasionally noticed according to the model‐based estimation, which did not reflect our actual observation. Given that the models fit well (*R* ^2^ ≥ 95%), we attribute this discrepancy to the difficulties in rating at the early season due to tiny size of the lesions, which complicates their differentiation from lesions caused by *Pseudomonas syringae* pv. *aptata*, the causal agent of bacterial leaf spot disease in sugar beet. With the disease progressing, more distinguishable CLS features contributed to a more accurate rating.

In 2023, additional non‐inoculated control plots were included to determine the relative loss in WSY. We further compared the relative loss with AUDPC and spore quantity from each variety in both locations. Despite the relatively low AUDPC in variety C and its resistant property, we did not observe a significant lower relative loss of WSY in both locations compared to susceptible varieties. In contrast, the lowest AUDPC for variety D in both locations was consistent with the significantly low relative loss of WSY. We anticipated that these two highly resistant varieties may have different resistant mechanisms against *C. beticola*. By comparing the spore quantity and the relative loss of WSY, it seems likely that the overall spore flight intensity can somehow hint the relative yield loss as shown in location Marienstein. However, when the overall infestation pressure is too high, the overall spore flight intensity will not serve as an ideal parameter which can directly reflect yield loss of varieties bearing various resistant properties.

Our study aimed to investigate the impact of variety resistance on the disease epidemiology of CLS with particular focus on the spore flight. In the past few decades, several studies have been conducted by using spore samplers to research on the aerobiology of various plant fungal pathogens.^40^ In our study, Roto Spore Samplers were used to capture the air‐dispersed spores from the plots grown with different varieties. Sampling of the spore‐embodied rods was performed every week, together with disease monitoring. As a result, we were able to demonstrate the spore flight intensity of each variety in different years and trial locations. This method was a solid, pre‐developed method which enables scientists in this field to quantify air‐borne spores of *C. beticola*. In addition to the advantage of adjustable measuring intervals,^4^ easy DNA extraction without needing additional spore germination step simplifies the sampling process. However, we were not able to detect spores before the occurrence of symptoms, which is aligned with the study of Imbusch *et al*.,^4^ who used the same device for spore detection.

The results in spore flight monitoring over 2 years conveyed the different capacity in producing airborne spores among varieties. The first instance of spore detection in our study was 7 and 5–6 wpi in 2022 and 2023, respectively. A study performed in the United States to detect *C. beticola* aerial spores with a volumetric spore trap reported the first detection of spores 1 month after inoculation,^8^ which aligns with our study. In addition, aerial spores were only detectable when certain DS was reached. In our study, the start of spore detection was when the average DS was above 10%. In both years, we observed a significantly less intensive spore flight from resistant varieties. Furthermore, the spore flight intensity in all varieties growing in the same locations followed a similar temporal pattern, indicating that higher variety resistance does not delay the timing of sporulation and spore dispersal. These results suggest that highly resistant varieties can reduce aerial spore production of *C. beticola*, resulting in less severe symptoms later in the season. This means that a smaller *C. beticola* population will be exposed under fungicide application for resistant varieties in practice, which can result in delayed development of fungicide resistance. Moreover, a positive correlation between cumulative spore quantity and DS in our field trials suggested that the spore flight intensity can be estimated by the disease development. This finding aligned with the study from Imbusch *et al*.,^4^ in which a positive correlation between these factors were found as well. The positive correlation between airborne spore quantity and DS is not restricted in the pathosystem of CLS. A study conducted in Belgium on wheat leaf rust investigated the correlation between the airborne spore quantity of its causal agent *Puccinia triticina* and the DS, in which a significantly positive correlation between these two factors was reported as well.^41^

In the pathosystem of CLS and sugar beet it remains to be revealed, whether the reduced aerial spores in resistance varieties is caused by spore deposition, spore germination or other following infection process. Other studies reported a lower germination rate of fungal pathogen spores on certain host varieties of peanut and barley.^42^, ^43^ Hence, an additional glasshouse inoculation assay was performed intended to investigate the sporulation capacity of each single lesion in our four varieties‐of‐interest. Despite the distinct resistant properties, we observed no significant differences in the amount of fungal DNA in single lesions from these varieties. We assume, the quantity of fungal DNA determined in our assay can be a reliable parameter indicating the fungal biomass. Therefore, these results suggest that the lower spore production observed in our field trails from resistant varieties was probably attributed to the lower DS and not caused by lower sporulation capacity per lesion. Spore quantification by counting was yet challenging since detached fungal material contains a mixture of complete conidia, conidial fragments, as well as mycelial fragments. This will further lead to the difficulty in distinguishing between true conidia and mycelial fragments due to the morphological traits of *C. beticola* conidia.

To sum up, our findings provide a comprehensive insight into the epidemiology of CLS in relation to variety resistant properties. In addition, it is the first study demonstrating that variety resistance can contribute to reduced airborne inoculum. This is especially important in regions with a high proportion of sugar beet in the crop rotation. Moreover, it shows that the progress in breeding has increased the resistance against CLS which can also contribute to a reduced fungicide application in sugar beet.

## 5 CONCLUSION

By monitoring the CLS disease development on sugar beet varieties bearing various resistance properties, we observed that resistant varieties showed delayed disease onset and reduced severity of symptoms. These observations were further confirmed in our variety specific models where resistant varieties showed delays in reaching certain DI and DS thresholds. Our results in spore flight detection and quantification revealed different varieties have different capacity in producing airborne spores. Positive correlations between cumulative DNA quantities detected from *C. beticola* aerial spores and the DS pinpointed that aerial spore flight intensity could reflect the resistant property of each variety.

## CONFLICT OF INTEREST STATEMENT

The authors have no conflicts of interest to declare.

## Supporting information


**Figure S1.** Roto spore sampler. (a) A sampler settled in the field. (b) Internal structure of a spore sampler.

**Table S1.** Fungicide application of control plots in 2023.

**Table S2.** Details of variety‐specific disease development models.

## Data Availability

Supporting documents are included within the article and its additional files. Additional data that support the findings of this study are available from the corresponding author upon reasonable request.
