# Supplementary figures and images for: Effect of sugar beet variety resistance on the disease epidemiology of Cercospora beticola
Source: Pest Manag Sci. 2025 Jan 23;81(6):2970–80. doi: 10.1002/ps.8666 (PMC12074627; doi:10.1002/ps.8666)

| **(a)**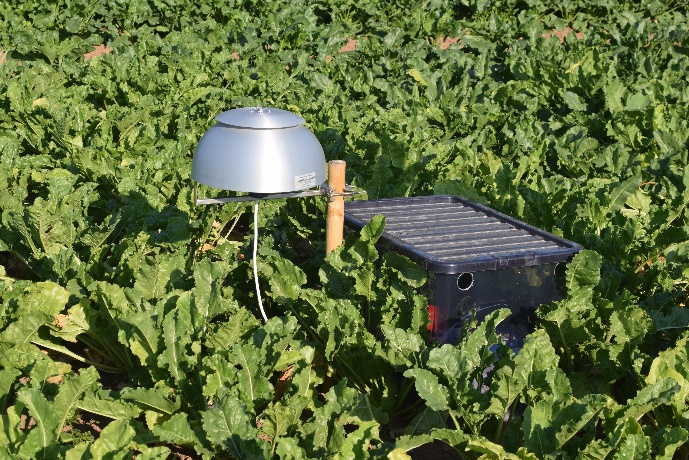 | **(b)**  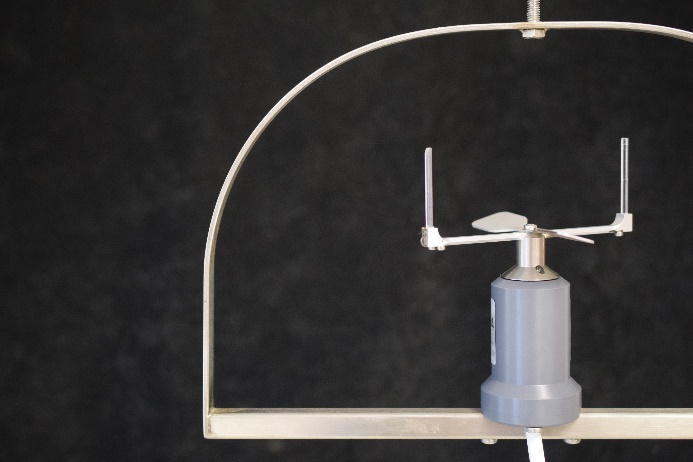 |
| --- | --- |

***Figure sup****. a). A spore sampler in the field. b). Inner structure of a spore sampler.*

Supplement: Supplementary file 1 — Figure S1. Roto spore sampler. (a) A sampler settled in the field. (b) Internal structure of a spore sampler. [file PS-81-2970-s001.docx]
