# Supplementary material for: Effect of sugar beet variety resistance on the disease epidemiology of Cercospora beticola
Source: Pest Manag Sci. 2025 Jan 23;81(6):2970–80. doi: 10.1002/ps.8666 (PMC12074627; doi:10.1002/ps.8666)
Supplement: Supplementary file 2 — Table S1. Fungicide application of control plots in 2023. [file PS-81-2970-s002.docx]

**TABLE S1** Fungicide application of control plots in 2023

| **Application time** | **Date** | **Fungicide product** | **Active ingredient** | **Concentration (g/L)** | **FRAC group** | **Dosage (L/ha)** |
| --- | --- | --- | --- | --- | --- | --- |
| 1^st^ | 11/07 | Propulse® | Fluopyram | 125 | 7 | 1.2 |
|  |  |  | Prothioconazol |  | 3 |  |
| 2^nd^ | 10/08 | Diadem® | Fluxapyroxad | 50 | 7 | 1.0 |
| 3^rd^ | 04/09 |  | Mefentrifluconazole | 100 | 3 |  |
